# A Telehealth-Based Behavioral Intervention for Cancer-Related Cognitive Decline in Older Adults Undergoing Systemic Therapy for Breast Cancer: Development and Usability Testing

**DOI:** 10.2196/85140

**Published:** 2026-01-28

**Authors:** J MacLaren Kelly, Lucia Berkhof, Oscar Y Franco-Rocha, Lewis Mustian, Valerie Targia, Jessica Bauer, Jessica Mortimer, Grace DiGiovanni, Daniel Millstein, Kassandra Scioli, Heidi DAurizio, Lauren DeCaporale-Ryan, Supriya Mohile, Michelle Janelsins, Robert J Ferguson, Alissa Huston, Allison Magnuson

**Affiliations:** 1 Department of Psychiatry, University of Rochester Medical Center, Rochester, NY, United States; 2 Roberts Wesleyan University, Rochester, NY, United States; 3 James P. Wilmot Cancer Institute, University of Rochester Medical Center, 601 Elmwood Avenue, Box 601, Rochester, NY, 14624, United States, 1 585-275-5823; 4 Patient Advocate Research Collaborator Group, SCOREboard, Rochester, NY, United States; 5 College of Osteopathic Medicine, University of New England, Biddeford, ME, United States; 6 Departments of Psychiatry and Neurology, Massachusetts General Hospital, Harvard Medical School, Boston, MA, United States; 7 Health Services, University of Waterloo, Waterloo, ON, Canada; 8 Department of Surgery, University of Rochester Medical Center, Rochester, NY, United States; 9 Department of Medicine, University of Rochester Medical Center, Rochester, NY, United States; 10 St Jude Children’s Research Hospital, Memphis, TN, United States

**Keywords:** geriatric oncology, older adults, telehealth or videoconferencing, cognition, cancer-related cognitive decline

## Abstract

**Background:**

Cancer-related cognitive decline (CRCD) is a significant problem; interventions are needed to mitigate CRCD for older adults (aged ≥65 years).

**Objective:**

Our objective was to develop and evaluate the usability of Memory and Attention Adaptation Training–Geriatrics (MAAT-G), a CRCD intervention for older adults with breast cancer undergoing systemic treatment.

**Methods:**

We conducted an intervention adaptation study to develop MAAT-G. MAAT-G is a cognitive behavioral therapy–based intervention delivered by a health professional over the course of 10 weekly individual workshops via videoconferencing. To develop MAAT-G, the contextual, cohort-based, maturity, and specific challenge framework was used for preliminary adaptations. Patient advocate collaborators guided further refinement, reviewing MAAT-G workshop content, the participant workbook, and intervention delivery via videoconferencing to optimize relevance and usability for older adults. The usability of MAAT-G and its videoconferencing delivery were subsequently evaluated in 4 older adults with breast cancer using the System Usability Scale (score range 0‐100; >67 being above average) and through semistructured qualitative interviews.

**Results:**

Numerous adaptations were made to address the unique needs of older patients using the contextual, cohort-based, maturity, and specific challenge framework and patient advocate feedback. Usability testing included 4 female patients with breast cancer (mean age 73.3, SD 3.77; range 67-77 years). Patients were receiving systemic therapy (2 receiving adjuvant therapy and 2 receiving advanced-stage disease therapy). One patient had an educational level lower than high school; 3 had some college education or higher. All 4 patients completed study procedures, including 10 MAAT-G workshop sessions (100% intervention adherence). The mean System Usability Scale score was 90.6 (SD 13.51), indicating good usability.

**Conclusions:**

MAAT-G is a behavioral intervention developed to mitigate CRCD. It is designed specifically for older adults and showed above-average usability in this population.

## Introduction

Cancer-related cognitive decline (CRCD) is a significant problem. Up to 75% of patients receiving active cancer treatment experience CRCD, which may manifest as difficulties in attention, processing speed, executive function, and memory [1-3]. In addition to CRCD, older adults with cancer (aged ≥65 years) also experience age-related cognitive decline, further compromising their cognitive health [4-8]. For instance, half of older adult women receiving adjuvant chemotherapy for breast cancer report worsening of cognition, and 25% have measurable declines on neuropsychological testing 6 months after chemotherapy [9,10]. CRCD can also compromise the functional independence of older adults, such as the ability to remain independent with managing medications or finances (eg, activities of daily living) [11]. Thus, addressing the cognitive needs of older adults with cancer is essential to promote independence, enhance their well-being, and promote healthy aging. However, CRCD interventions tailored to them do not exist [12].

Memory and Attention Adaptation Training (MAAT) is a cognitive behavioral therapy (CBT)–based intervention for CRCD that focuses on an individual’s psychological response to injury as compared to the biological events triggering CRCD [13]. MAAT has 8 manualized workshops supplemented by a participant workbook and is individually delivered by a psychologist via videoconferencing. Together, the workshops and workbook provide instruction and practice with adaptive behavioral coping skills, stress management techniques, and compensation strategies to manage CRCD symptoms [14]. Although MAAT has been associated with greater scores on perceived cognition functioning, verbal memory, and processing speed in 3 separate studies, these populations were primarily middle-aged survivors of breast cancer who completed treatment months before enrolling in the intervention [13-15]. Thus, additional research is necessary to examine the effectiveness of MAAT in other populations, such as older adults with cancer. However, adapting the intervention to their specific needs and resources is essential to increase their usability and potential benefits.

Older adults are more likely to be diagnosed at later cancer stages [16] that may require extended treatments. Furthermore, due to the increased risk of age-related cognitive decline and CRCD, adapting MAAT to intervene earlier (eg, concurrent with cancer therapy) has the potential to improve outcomes in this vulnerable population. Thus, the goals of this study were to (1) adapt MAAT to the unique needs of older adults with breast cancer (eg, develop Memory and Attention Adaptation Training–Geriatrics [MAAT-G]) and (2) evaluate the acceptance and usability of MAAT-G in a sample of older adults with breast cancer receiving systemic therapy.

## Methods

### Overview

We conducted an intervention adaptation study to develop MAAT-G. Specifically, MAAT-G was developed in two phases: (1) preliminary adaptations based on the contextual, cohort-based, maturity, and specific challenge (CCMSC) framework [17]; and (2) refinement with collaboration of patient advocates. We then conducted an evaluation of the usability of MAAT-G and its telehealth delivery model in a single-arm study of older adults receiving systemic therapy for breast cancer.

### MAAT-G Adaptation

#### Preliminary Adaptation

The CCMSC model [17] is informed by research on aging and older adult social contexts and has been previously used to adapt CBT-based interventions [18,19]. To optimize the relevance and usability of MAAT for older adults, study authors (AM, RJF, LD-R, and DM) made initial adaptations based on CCMSC model principles as follows:

- Contextual: identification of the unique social and environmental factors that influence the targeted population, such as considering the patient’s living situation (eg, retirement communities, aging services, and long-term care facilities), community infrastructure, social support network and role changes
- Cohort-based: analysis of specific factors of the population of interest, such as their cognitive abilities, educational level, word use, normative life paths, and social-historical life experiences, as well as considering the fact that older adults may identify with an aging cohort with specific beliefs and attitudes and unique needs (eg, additional technological support)
- Maturity: consideration of factors associated with aging, such as potential preexisting conditions (eg, comorbidities) and cognitive and emotional complexity, as well as analyzing how family and life experiences may influence perspectives, expertise, areas of competence, and accumulated interpersonal skills
- Specific challenge: examining conditions or situations that may create barriers to engagement, such as chronic illnesses, disabilities, and age-related conditions (eg, hearing loss and visual impairment), as well as additional exploration of social challenges associated with aging, such as grieving for loved ones and requiring caregivers

#### Additional Refinement

Following the initial adaptations, study authors (AM and GD) presented the protocol to a local older patient advisory board (Stakeholders for Care in Oncology and Research for Our Elders Board [SCOREboard]) with experience in the design of clinical trials for older adults with cancer [20-23]. Authors collected high-level feedback from the full patient advisory board and made initial modifications as necessary. Next, study authors met with 2 SCOREboard members (LM and VT) to conduct an in-depth review of MAAT-G and its delivery method. Four meetings were conducted, each focusing on a separate aspect of MAAT-G; workshop content (across 2 meetings); participant workbook content, presentation, and formatting; and aspects of the intervention (eg, technology interface and development of instructions and support materials). The meetings lasted between 1 and 2 hours, and SCOREboard members reviewed materials before and in between meetings.

### Assessment of Usability and Delivery Mode

#### Eligibility

Following the adaptations, we tested the usability of MAAT-G in an open single-arm study. To be included in the usability phase, participants had to (1) be aged ≥65 years, (2) be diagnosed with breast cancer (any stage), (3) be receiving systemic therapy with at least 2 cycles remaining, (4) be able to speak and read English, and (5) have decision-making capacity. We focused on patients aged ≥65 years as they experience the highest rates of breast cancer incidence and are more likely to experience CRCD, and there are no existing interventions focusing on their unique needs [24].

#### Recruitment

Patients were recruited from the Comprehensive Breast Cancer Center at the Wilmot Cancer Institute in Rochester, New York. The study team obtained approval to screen clinic schedules using electronic medical records for prospective patients. Following the identification of a prospective patient, the study team contacted the patient’s primary oncologist to inquire about the patient’s decision-making capacity for informed consent and obtain approval for approaching the patient about study participation. The study staff next approached patients at the clinic to present the study, answer study questions, and obtain informed consent.

#### Usability Assessment

We assessed the usability of MAAT-G quantitatively using the System Usability Scale (SUS) [25,26]. Possible scores range from 0 to 100, with a score greater than 67 indicating average or good usability [27,28]. This threshold was used as the benchmark for determining usability in this study. We also examined the usability qualitatively through semistructured interviews. The semistructured interviews consisted of 4 questions on patients’ experience over the course of the study regarding the workshop content, the use of the tablet, the participant workbook, and the potential impact of MAAT-G. All interviews were conducted by 1 staff member not involved with intervention administration. All interviews were also audio recorded to generate a transcript.

### Procedures

Following informed consent, we provided patients with a bound copy of the patient workbook, a tablet with a HIPAA (Health Insurance Portability and Accountability Act)-compliant videoconferencing application, and a set of instructions on the use of the tablet. The intervention took place over the course of 10 to 12 weeks and consisted of 10 MAAT-G workshops delivered one-on-one. One member of our study staff (psychology postdoctoral fellow) delivered all workshop sessions after receiving appropriate training. Each workshop session was delivered over videoconferencing approximately once per week. All intervention sessions were video recorded; videos were then reviewed by the principal investigator to ensure fidelity to the intervention delivery protocol.

### Data Analysis

To assess the usability of MAAT-G, we summed the responses on the SUS for each participant and then computed a mean value across patients [25,26]. All quantitative analyses were conducted using SPSS (version 28; IBM Corp). Qualitative interview transcripts were imported into the MAXQDA software (VERBI GmbH) for sorting, coding, and analysis. The principal investigator and a senior staff member (both with prior experience in qualitative data analysis) independently read the transcripts to familiarize themselves with the data. They then met to jointly code the transcripts using an inductive approach; any coding discrepancies were discussed until consensus was reached. We opted for a joint coding process considering the low number of transcripts. Emerging codes were then grouped into categories based on similarity, and categories were grouped into themes, consistent with the content analysis approach [29].

Finally, quantitative and qualitative data were compared against each other to examine how the SUS scores aligned with the perceived intervention experience. These results were used to determine whether additional intervention refinements were necessary and, if so, guide such modifications.

### Ethical Considerations

The Institutional Review Board of the University of Rochester oversaw and approved the study (STUDY00003900). All participants provided informed consent before study enrollment and data collection. The information provided by the participants was deidentified and kept on a secure, password-protected platform. Participants received US $30 per workshop session they completed. The authors confirm that participants were made aware of and provided informed consent for the publication of the study findings and example quotes.

## Results

### MAAT-G Adaptation

As shown in Table 1, the preliminary adaptations comprised a reduction in the quantity of material covered in each session. This helped reduce the quantity of information provided in one sitting, concordant with CCMSC principles. However, this adaptation subsequently necessitated an extension in the number of workshops from 8 to 10.

**Table 1.** Examples of Memory and Attention Adaptation Training–Geriatrics (MAAT-G) adaptations implemented through the contextual, cohort-based, maturity, and specific challenge (CCMSC) model and stakeholder feedback.

CCMSC^a^ component	Preliminary adaptation conducted by expert investigators	Intervention refinements based on patient advocates’ (SCOREboard^b^) input
Contextual factors	Content experts added a section to the MAAT-G interventionist manual on social context factors and how they may influence compensatory strategies for older adults Added a screening question to the MAAT-G interventionist manual to assess social support to encourage leveraging social support resources for compensatory strategies	Case scenarios were updated to be more age relevant (eg, modification in occupations: removing “work” and replacing it with “volunteering” or “household chores”)
Cohort-based factors	Added a section to the interventionist manual on cohort-based factors and their potential influence on coping mechanisms and stress responses Modified the patient workbook by minimizing complex terminology to accommodate potentially lower educational levels	Reviewed workbook changes and suggested logistical modifications (eg, using thick paper to print the workbook in case the patient was experiencing neuropathy) Developed technology support materials to enhance inclusiveness of those with limited fluency with technology
Maturity factors	Added a section to the interventionist manual on maturity factors and their effect on pace of information receipt, coping mechanisms, and stress response Extended the workshop number from 8 to 10 to decrease the amount of material in each workshop Reduced complexity of examples in patient manual	Updated the intervention materials to be sensitive to patients living alone (eg, widowed); highlighted the relevance of acknowledging these factors to the intervention Suggested providing materials (eg, stylist and stand) to facilitate engagement during the intervention sessions
Specific challenge factors	Added a step to the interventionist manual to screen for hearing loss, allowing the interventionist to proactively adapt volume and rate of speech while delivering the intervention Adjusted font type and size (eg, Aptos body, 15 points, double spaced) in the patient workbook to accommodate potential visual impairments	Checking the technological devices provided to facilitate their use (eg, step-by-step guide to access the Zoom application) Flexibility in delivery and scheduling to account for potential health challenges (eg, having sessions in the evening instead of early in the morning and a flexible window between sessions to adapt to the patients’ calendars)

a We followed the CCMSC model as determined by Knight [17] and with involvement of patient advocate collaborators.

b SCOREboard: Stakeholders for Care in Oncology and Research for Our Elders Board.

The patient advocates reviewed each adaptation based on CCMSC components and confirmed the appropriateness of the modifications. Most of the additional feedback provided by the patient advocates centered on logistical adaptations to improve the intervention delivery (eg, the type of paper needed to print the workbook and development of a technology user’s manual) and refinement of case examples to ensure their relevance (eg, removing wording associated with work and employment and replacing it with other occupations, such as household chores or volunteering). Furthermore, patient advocates (SCOREboard members) played a key role in the development of robust technology support materials to which patients could refer if questions arose during the study. The materials described basic operations such as how to log into the tablet, how to charge it, and how to log into the Zoom application—including photographs of steps in addition to text instructions. Different versions were developed to accommodate the participants’ technological needs (eg, patients who received a data-enabled vs Wi-Fi–only tablet). The content of the adapted intervention, MAAT-G, is shown in Table 2.

**Table 2.** Content overview of the adapted intervention: Memory and Attention Adaptation Training–Geriatrics (MAAT-G).

Session number	Title	Overview
1	Introduction	Presentation of MAAT-G Education on the impact of cancer and its therapy on memory and attention Importance of self-monitoring memory and attention and strategies
2	Relaxation skills	Review of session 1 materials and skills Education on the impact of stress on the body Strategies to manage stress (progressive muscle relaxation and brief relaxation)
3	Self-instructional teaching	Review of session 2 materials and skills Memory and attention adaptation skills (self-instructional training, verbal and “silent” rehearsal skills, and rhythmic skills)
4	Cognitive flexibility	Review of session 3 materials and skills Education on cognitive flexibility Skills to increase cognitive flexibility (probability re-estimation and decatastrophizing)
5	Keeping a schedule and memory routines	Review of session 4 materials and skills External skills to assist with memory and attention problems (scheduling and memory routines)
6	External cueing and distraction reduction	Review of session 5 materials and skills Education on external cues to assist with memory and attention Strategies to help with distraction reduction
7	Activity scheduling and pacing	Review of session 6 materials and skills Education on the importance of scheduling and pacing Strategies to assist with scheduling and pacing (active listening, summarization, and clarification)
8	Fatigue management and sleep quality	Review of session 7 materials and skills Education on fatigue and sleep improvement Skills for managing fatigue (activity pacing, relaxation skills, and exercise and diet) Skills for sleep quality improvement (sleep hygiene and exercise and diet)
9	Visualization skills	Review of session 8 materials and skills Education on visualization skills Visualization skills
10	Tying it all together	Review of materials and skills covered in the workshops Tying together all MAAT-G skills learned Development of strategies to maintain the use of the skills learned

### Assessment of Usability

#### Overview

A total of 4 patients were approached to participate in the usability assessment of MAAT-G. All consented to participate, suggesting preliminary acceptance of recruiting older adults with breast cancer receiving systemic therapy. All participants also completed all study procedures, including 10 MAAT-G workshop sessions, without delay (100% intervention adherence).

#### Sample

The clinical and demographic characteristics of the participants included in the usability phase are shown in Table 3. Briefly, all participants were women with breast cancer. The mean age was 73.3 (range 67-77) years, and all identified as White individuals. Two of the patients were married and currently lived with their spouses, whereas 2 were widowed and lived alone. Annual household income ranged from less than US $20,000 to over US $100,000. All patients were enrolled in Medicare. Two patients were presently employed (1 part time and 1 full time). All but 1 participant (who had an educational level lower than high school) had at least some college education. All patients were also receiving active systemic therapy at the time of study participation (2 were receiving adjuvant chemotherapy–based regimens, and 2 were receiving advanced-stage disease regimens with cyclin-dependent kinase 4 or 6 inhibitor therapy).

**Table 3.** Clinical and demographic characteristics of phase 2 participants.

	ID01	ID02	ID03	ID04
Age (y)	77	67	74	75
Race and ethnicity	White; ethnicity not reported	Non-Hispanic White	Non-Hispanic White	Non-Hispanic White
Marital status	Widowed	Married	Married	Widowed
Living situation	Living alone	Living with spouse	Living with spouse	Living alone
Income (US $)	>20,000	<100,000	Not reported	Between 50,000 and 100,000
Health insurance	Medicare	Medicare	Medicare	Medicare
Employment status	Retired	Employed full time	Homemaker	Employed part time
Highest educational level	9th-11th grade	Junior college degree	Some college	Advanced degree
Cancer stage	Local	Local	Advanced	Advanced
Cancer treatment	ACT^a^	ACT	CDK^b^ 4 or 6 with aromatase inhibitor	CDK 4 or 6 with aromatase inhibitor
System Usability Scale score	100	67.5	100	95

a ACT: adriamycin, cyclophosphamide, and taxol.

b CDK: cyclin-dependent kinase.

The session delivery audit conducted by the principal investigator did not suggest significant deviations from the administration protocol. Thus, fidelity to the protocol was considered high.

#### Usability

Quantitatively, we found that the mean total score on the SUS was 90.6 (SD 13.51; range 67.5‐100), suggesting above-average usability [25,26] and exceeding our a priori usability threshold (>67). Although the lowest score was also higher than the a priori usability threshold, we paid special attention to the qualitative data from this participant to examine specific recommendations. However, the qualitative data were consistent between participants regardless of usability score. The content analysis yielded 4 themes (technology support, comfort with technology, experience with the intervention, and utility of its content) and suggested areas of further modification (Table 4).

**Table 4.** Qualitative themes and example quotes from interviews on usability.

Qualitative theme	Example quotes from participants
Technology support	“It worked out really well. The only time I didn’t know where to push the volume; I didn’t know where that was. I found it to be a great tool. I found the information you gave me on how to set it up, how to get started with Zoom, it was simple to operate it by reading the pamphlet you gave me. I found that—it made the class easy without guessing.” “I thought having your phone number, the one time we couldn’t get in to him [interventionist] for some reason—I’m not sure why—but we had your phone number and we were able to make contact right away. That was really good.”
Comfort with technology	“The iPad was very easy to use, much easier than the computer.”
MAAT-G^a^ experience	“Yes, it was useful. I was pretty diligent; I did my homework every week. As a matter of fact, I read a little ahead of time because I liked to be a little familiar. We took notes.” “I felt very little anxiety looking for something. Usually my anxiety kicks in. I just relaxed myself for a little bit and then I remembered where I put my glasses after a few minutes of doing that.” “Basically, the whole experience was great.” “I think it was the right number of sessions.”
Areas for further MAAT-G modification	“The only thing I did not like about it is the paper, you can’t get it to write on it with pencil, it doesn’t show.” “...putting page numbers on the book.”

a MAAT-G: Memory and Attention Adaptation Training–Geriatrics.

Overall, the patients found the technological support manual and staff guidance to be useful for learning how to operate the tablet. Furthermore, it was helpful to reach the study staff via phone for technological support in joining the videoconference session or operating the tablet. Over the course of the study, patients reported increased comfort with the tablet. The participants also described that the material covered during the workshops was useful. There were no additional suggestions on intervention content or delivery methods. Instead, participants’ suggestions centered on the workbook materials. Specifically, they suggested printing the workbook on nonglossy paper so that they could take notes in the margins, as well as adding page numbers to the workbook to easily locate different sections. These 2 logistical suggestions were incorporated into our current randomized controlled trial. Other intervention aspects did not change.

## Discussion

We adapted a CBT-based intervention to address CRCD symptoms among older adults with cancer undergoing systemic therapy and examined its usability and acceptability. MAAT-G engages participants in a 1:1 telehealth setting to explore and learn coping skills, stress management techniques, and methods to target episodes of CRCD [1]. We strove to promote universal usability and acceptance using telehealth and early and meaningful involvement of patient advocates throughout the intervention adaptation process.

Although several studies have evaluated behavioral interventions to address CRCD symptoms, their scope is limited [30]. Similar to MAAT-G’s parent intervention (MAAT) [13-15], most existing interventions focus on younger survivors of cancer and individuals who have completed treatment months to years before enrollment. For instance, Cherrier et al [31] studied the effect of a 7-week workshop among middle-aged (mean age 58.9 years) people with breast, bladder, prostate, colon, and uterine cancer with an average of 4.84 years after treatment completion. Similarly, ReCog, a group cognitive rehabilitation intervention, improved perceived cognitive functioning in survivors of cancer with an average of 3 years since treatment completion [32]. Our findings expand the current literature by providing a CRCD intervention for older adults undergoing treatment and showing preliminary evidence of its usability.

MAAT-G was designed as a telehealth delivery model given that older patients in active cancer treatment often have frequent in-person appointments. Thus, having additional in-person study visits may create accessibility barriers for those no longer driving independently. This design was based on other studies demonstrating that telehealth provides participants with the flexibility and comfort of completing all study-related tasks from home [14]. Additionally, a one-on-one intervention delivery model such as that of MAAT-G was preferred due to its scheduling flexibility, which allows sessions to be coordinated around existing clinic and therapy appointments.

Previous studies have demonstrated that the inclusion of patient advocates as research collaborators has significant benefit in clinical trials [33,34]. For this study, we collaborated with the Cancer and Aging Research Group (CARing) SCOREboard, a patient advocate advisory board supported by the Cancer and Aging Research Group. CARing SCOREboard members provided critical insights about study design, aspects related to intervention adaptation, and guidance on technology support. The inclusion of the patient perspective throughout the study design and intervention adaptation led to more patient-focused study procedures and design, which contributed to the favorable usability of the resulting intervention and, ultimately, advocated for and prioritized the study participants’ needs.

The foundation of the MAAT-G intervention was developed using the CCMSC model [17], which has been previously used in other CBT adaptation studies for older adult populations. For example, Trevino et al [35] used the CCMSC framework to develop the Managing Anxiety From Cancer (MAC) intervention, which addresses anxiety symptoms among older adults with cancer. Both the MAC and MAAT-G adaptations emphasize the cohort component of the CCMSC model, which highlights ideas such as membership and beliefs about and attitudes toward the problems. As such, MAC minimized the psychological language and developed examples with situations that older participants with cancer may experience. Similarly, MAAT-G revised the language of the intervention materials and created examples based on real-life experiences of older adults receiving cancer therapy. Despite this, the 2 interventions differed in some CCMSC components, particularly the specific challenge and maturity components. For example, MAAT-G accounted for the technological challenges of telehealth intervention delivery by adjusting the font size of the materials, developing a tablet manual, and screening for hearing loss, among others. Regarding the maturity components, MAC decreased the total number of sessions to minimize the participants’ time commitment. In contrast, MAAT-G increased the number of sessions to reduce the quantity of information delivered during each session.

This study had multiple strengths, such as the comprehensive, evidence-based adaptation approach; the intensive patient advocate involvement; and the patient usability and engagement rates. Allowing patients to provide direct input on their experience helped the research team prioritize intervention components and delivery factors and overall tailor the intervention based on input from the targeted population. Furthermore, the technological support manual allowed older adults to feel more comfortable with the technological components of the intervention over time. Thus, our findings highlight activities that can promote technological engagement among older adults, which have the potential to improve the representation of this population group in future technology-based clinical trials.

A limitation of this study is the small sample size of the usability phase. However, our sample size is congruent with those of other usability studies in the field [36]. Furthermore, there was low variability in the clinical and demographic characteristics of the participants (all were recruited from a single cancer center, identified as non-Hispanic White individuals, and were older women undergoing systemic treatment for breast cancer but healthy enough to complete the intervention, and most of them also reported high educational levels), so generalizability to other populations of older adults, particularly those most often affected by CRCD and digital barriers, is limited, and further adaptations of the intervention might be required for those populations. Finally, capacity for study participation was confirmed by the patients’ treating oncologists; future studies could consider a more standardized method for capacity assessment for participation.

In summary, we developed MAAT-G, a CBT-based intervention to address CRCD symptoms among older adults receiving active treatment for breast cancer and showed its preliminary usability and acceptability in a small cohort. A comprehensive, evidence-based, and patient-informed approach was followed, which improved accessibility, usability, and engagement. The feasibility and preliminary efficacy of MAAT-G are currently being evaluated in a pilot randomized clinical trial testing its effects on CRCD symptoms among older adults undergoing systemic breast cancer treatment.

## Abbreviations

CARing: Cancer and Aging Research Group
CBT: cognitive behavioral therapy
CCMSC: contextual, cohort-based, maturity, and specific challenge
CRCD: cancer-related cognitive decline
HIPAA: Health Insurance Portability and Accountability Act
MAAT: Memory and Attention Adaptation Training
MAAT-G: Memory and Attention Adaptation Training–Geriatrics
MAC: Managing Anxiety From Cancer
SCOREboard: Stakeholders for Care in Oncology and Research for Our Elders Board
SUS: System Usability Scale
